# Evaluating the Intestinal Immunity of Asian Seabass (*Lates calcarifer*, Bloch 1790) following Field Vaccination Using a Feed-Based Oral Vaccine

**DOI:** 10.3390/vaccines11030602

**Published:** 2023-03-06

**Authors:** Thanusha Raju, Tilusha Manchanayake, Amir Danial, Mohd Zamri-Saad, Mohammad Noor Amal Azmai, Ina Salwany Md Yasin, Norhariani Mohd Nor, Annas Salleh

**Affiliations:** 1 Aquatic Animal Health and Therapeutics Laboratory (AquaHealth), Institute of Bioscience, Universiti Putra Malaysia, Serdang 43400, Selangor, Malaysia; 2 Department of Veterinary Laboratory Diagnosis, Faculty of Veterinary Medicine, Universiti Putra Malaysia, Serdang 43400, Selangor, Malaysia; 3 Department of Biology, Faculty of Science, Universiti Putra Malaysia, Serdang 43400, Selangor, Malaysia; 4 Department of Aquaculture, Faculty of Agriculture, Universiti Putra Malaysia, Serdang 43400, Selangor, Malaysia; 5 Department of Veterinary Preclinical Sciences, Faculty of Veterinary Medicine, Universiti Putra Malaysia, Serdang 43400, Selangor, Malaysia

**Keywords:** *Vibrio*, vibriosis, vaccine, GALT, Asian seabass, aquaculture

## Abstract

This study describes the levels of gut lysozyme and IgM, the number, size and density of gut-associated lymphoid tissue (GALT) regions, and the lymphocyte population in Asian seabass following field oral administration of a feed-based vaccine. Fish in a grow-out farm were selected and divided into two groups; Group 1 was vaccinated at week 0, 2, and 6, while Group 2 was not vaccinated. Samplings were done at 2-week intervals when the fish were observed for clinical signs, and gross lesions were recorded. The intestinal tissue and gut lavage fluid were collected. GALT regions (numbers, size, density and population of lymphocytes) were analyzed. Clinical signs such as abnormal swimming pattern and death, and gross lesions including scale loss, ocular opacity, and skin ulceration were observed in both groups. At the end of the study, the incidence rate between both groups were significantly different (*p* < 0.05). The gut IgM level and lysozyme activity, lymphocyte population, number, size and density of GALT regions of Group 1 were significantly (*p* < 0.05) higher than Group 2. Therefore, this study concludes that the feed-based vaccine reduces the incidence of vibriosis by stimulating the gut immunity of the vaccinated fish with an enhanced GALT region, specific IgM production against *Vibrio harveyi,* and lysozyme responses.

## 1. Introduction

*Vibrio* spp. are Gram-negative bacteria, with a sheath-enclosed polar flagella that are either straight or comma-shaped rod [1]. They are found mainly in the marine environment [1,2], but despite being halophiles, they are also present in freshwater. *Vibrio anguillarum, V. ordalii, V. salmonicida, V. vulnificus, V. alginolyticus, V. harveyi, V. parahaemolyticus, V. ponticus* and *Photobacterium damselae subsp. damselae* are recognised as important species that cause vibriosis in cultured marine fishes [3,4]. Infection starts either from the intestine after oral ingestion, or from aberrated skin and gills. The resulting septicaemia leads to lodging of the bacterium in vital organs like liver, spleen and kidney [5]. Marine fish and shellfish are frequently affected by vibriosis thus, is regarded as one of the most important diseases of wild and reared aquatic animals [6]. Vibriosis is economically important in marine fish culture, where fishes are predisposed to at least one *Vibrio* sp. [3,4]. Hitherto, vibriosis has a worldwide distribution [3]. 

Bacterial diseases cause significant economic impacts to the marine aquaculture industry [7]. The economic loss due to vibriosis is largely incriminated to the high cumulative mortality, which is about 40% [2]. A recent study in Malaysia disclosed that the average cost of vibriosis in Asian seabass (*Lates calcarifer*) cultured in floating marine cage-culture system was 0.24 USD/tail and with mortality, it costs 0.19 USD/tail. The cost of treatment was 0.05 USD/tail and diagnosis was 0.003 USD/tail [8]. Thus, Malaysia was reported to face a total loss of 7.4 million USD due to vibriosis in cultured marine fish [8]. In early 1990, Indonesia reported more than 100 million USD due to vibriosis in shrimp farm. Vibriosis was shown to reduce the growth rate in marine cultured fish due to stress and inappetence, thus causing decrease in productivity [2]. 

Antibiotics, particularly oxytetracycline, tetracycline, quinolones, nitrofurans, potentiated sulfonamides, trimethoprim, sarafloxacin, flumequine and oxolinic acid are used to control bacterial outbreaks, including vibriosis [2]. Although they are effective to control vibriosis, the risk of developing antibiotic resistance is a public health concern. Therefore, researches have been conducted to explore the use of vaccine against vibriosis in fish. Unlike antibiotics, which is bactericidal, vaccines stimulate the immune system of fish through antibody production that protects the fish from specific diseases [4,9,10]. 

In general, livestock vaccination should consider three main aspects; the ability to confer long-term protection, the ease of vaccine delivery, and widespread vaccine coverage [11]. Considering these aspects, oral vaccination is superior in the latter two aspects. However, the intraperitoneal and intramuscular injections could possibly result in better protection levels by eliciting systemic immune responses [12,13]. Nevertheless, vaccines delivered via injection have few disadvantages such as fish must be anesthetized prior to vaccination to reduce stress. Furthermore, it is time consuming, laborious, and may cause severe reaction, especially in the event where it was not properly administered [11]. 

Oral vaccination is the easiest delivery method and the antigen is directly delivered into the digestive system of the fish [14], stimulating the mucosal immunity through the mucosa-associated lymphoid tissue (MALT). In the intestinal tract of animals including fish, the gut-associated lymphoid tissue (GALT) is a MALT that is expected to be stimulated following oral vaccination. Studies pertaining to GALT is important as the intestine is a major portal of entry of vibriosis; commercial pellets are used to feed farmed fish thus, provides opportunity to regulate fish health by addition of drugs and vaccines [15].

Previously, we developed and tested a feed-based vaccine against vibriosis that was proven to be efficacious to prevent vibriosis in Asian sea bass and grouper in field settings [16,17]. Furthermore, it also resulted in significant increment of growth rate, decreased rate of *Vibrio* spp. isolation, and higher lysozyme activities and IgM in the mucus and serum [16]. Since this is a feed-based vaccine, it is expected to also stimulate the mucosal gut immunity. Thus, this study seek to understand the mechanisms of this feed-base against vibriosis by assessing the mucosal immune response of Asian seabass.

## 2. Materials and Methods

### 2.1. Feed-Based Vaccine Preparation

Trypticase soy agar (TSA) (Oxoid, UK) supplemented with 1.5% NaCl was used to culture *Vibrio harveyi* strain VH1. Then, the colonies were transferred into flasks containing Trypticase soy broth (TSB) (Oxoid, UK) with 1.5% NaCl and were incubated at 30 °C in a rotary shaker at 150 rpm overnight. The standard plate count technique was used to determine bacterial concentration before the concentration was adjusted to 6.7 × 10^8^ cfu mL^−1^. Then, 0.5% neutral-buffered formalin in phosphate-buffered saline (PBS) was added and incubated for 24 h at 4 °C to inactivate the bacteria. The suspension was washed four times using sterile PBS followed by centrifugation at 6000× *g* for 15 min to remove the media and formalin residues. The inactivated bacteria was then resuspended into sterile PBS, streaked onto TSA supplemented with 1.5% NaCl, and incubated at 30 °C overnight. This was done to reconfirm that the bacterial cells were successfully inactivated. 

A feed-based adjuvant vaccine was prepared following previously described method [18]. The palm oil (Vesawit, Malaysia) was used as an adjuvant and oil was mixed with the inactivated bacteria to a final concentration of 10%. To prepare the vaccine-incorporated vaccine feed, commercial fish feed pellets (Star Feed, Star Feed Mills SDN. BHD., Klang, Malaysia) were ground to a fine powder. The vaccine-adjuvant mixture was distributed onto the feed powder using an industrial mixture (Golden Bull-B10-A Universal Mixers, Johor Bahru, Malaysia) to achieve the final concentration of 10^8^ cfu g^−1^ of feed. PBS and palm oil were mixed with the same commercial pellet and was used as a sham feed-based vaccine. Finally, a mini feed pellet machine (Golden Avill, Guangdong Province, China) was used to re-pellet the vaccine-incorporated feed to the size of 1 × 0.5 cm before drying for 48 h at 30 °C. 

### 2.2. Fish

The study was conducted in a commercial farm in Pulau Ketam, Selangor, Malaysia. A total of 4800 Asian seabass with an average body weight of 182 ± 31 g were selected and kept within 8 net cages sizing 4 × 4 × 3.5 m. They were selected from a farm with endemic vibriosis and were kept in the same farm. Fifteen fish were randomly selected from each cage, and were dissected before swab samples were collected from the gut and kidney. The swabs were then cultured onto Thiosulfate-Citrate-Bile Salts-Sucrose (TCBS) agar as a selective media for *Vibrio* spp., followed by incubation for 24 h at 30 °C to ensure that the fish were free from *Vibrio* spp. As no *Vibrio* spp. was detected in these samples, the fish were determined to be free of vibriosis prior to the start of the study.

### 2.3. Experimental Design and Vaccine Regimen

**Figure 1:**
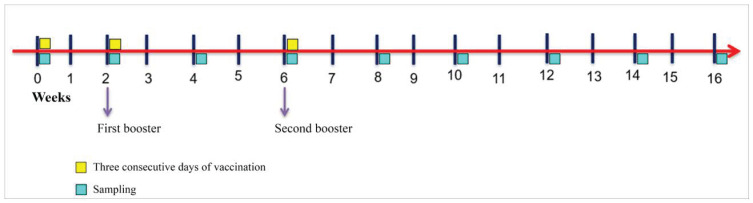
Summary of vaccination regimen using the feed-based inactivated *V. harveyi* vaccine in Asian seabass.

Fish of Group 1 were vaccinated by feeding with the feed-based inactivated vaccine, whereas Group 2 was fed with the sham feed-based vaccine and served as a non-vaccinated control group. All fish from both groups were not fed for 24 h prior to the feeding of feed-based vaccine for Group 1, and sham feed-based vaccine for group 2. This was to ensure intake of the feed for all fish. The first vaccination was done on week 0, followed by the first and second boosters on weeks 2 and 6, respectively. During each vaccination, the feed-based vaccine was given for three consecutive days at 4% body weight. On other days, the fish were fed with untreated commercialized pellets (Figure 1). The feeding and husbandry practices of the farm were maintained. The vaccination trial was carried out for a total of 16 weeks.

### 2.4. Sampling

Sampling was done at 2-week intervals starting from week 0 until the end of the study on week 16. During each sampling, data of incidence rates and general clinical signs were noted before 15 fish from each replicate were randomly selected and immediately euthanized by pithing. Necropsy was conducted immediately to examine for gross lesions. The gut was sampled for collection of lavage fluid [18] and histology examinations.

### 2.5. Incidence Rate

To determine the incidence rate, 15 random fish were collected from each replicate. Based on the presence of gross lesions suggestive of vibriosis, and positive *Vibrio* spp. isolation and detection, from the kidney and gut samples, the diagnosis were collected from 15 random fish of each replicate. These samples were subjected to bacteriology by culturing on TCBS agar for 24 h at 30 °C. Suspected colonies of *Vibrio* spp. were collected and confirmed by PCR using a previously described protocol [19]. Detection of *Vibrio* spp. in the kidney and gut were considered positive of vibriosis. The incidence rates were calculated using the following formula:

(1) (Number of fish with vibriosis/15)×100

### 2.6. Measurement of Gut Specific IgM against V. harveyi

The gut IgM levels were measured using indirect ELISA according to a previously described method, with minor modifications [18]. Flat-bottom microtitre plates (Greiner-bio One, Frickenhausen, Germany) were coated with 100 µL coating antigen containing 10^5^ formalin-killed whole cells of *V. harveyi* per mL, in carbonate-bicarbonate buffer per well. The plates were left overnight at 4 °C, followed by washing twice with phosphate buffer saline +0.05% Tween 20 (PBST). Then, 200 µL of 1% bovine serum albumin (BSA), diluted in PBS, was used to block the plate by incubation for 1 h at 37 °C. Once the reaction wells were washed thrice with PBST, 100 µL of 1:1000 gut lavage, diluted in PBS, was transferred into the reaction well and re-incubated for 1 h at 37 °C. The unbounded antibodies were removed by washing the reaction wells thrice with PBST. Specific IgM was detected using anti-Asian seabass IgM monoclonal antibody (Aquatic Diagnostics Ltd., Stirling, UK, 1/33 in PBS, 1 h), accompanied by incubation with anti-mouse HRP (1/5000, Nordic, 1 h). Then, 100 µL of TMB substrate solution (Thermo Fisher Scientific, Waltham, MA, USA) was added into the reaction wells after washing three time along with PBST. The reaction was stopped with 0.2 mol/L sulphuric acids. Multiskan spectrum microplate reader (Thermo Fisher Scientific Inc., Madison, WI, USA) was used to obtain the values by measuring the absorbance at 450 nm. The cut-off value was the highest possible true-positive rate that was used as an indication of protection. It was determined by performing ELISA on 100 samples collected from non-immunized and uninfected fish [20].

### 2.7. Measurement of Gut Lysozyme Activity

Lysozyme activity was measured based on the lysis of the lysozyme sensitive Gram-positive bacterium, *Micrococcus lysodiekticus* (Sigma-Aldrich, St Louis, MO, USA) [21]. Twenty-five µL of gut lavage fluid was added into 75 µL of lyophilized *M. lysodeikticus* cell suspension (Sigma, 75 mg/mL) prepared with 0.1 M phosphate citrate buffer at pH 6.3 in wells of a 96-well plate in triplicate. After rapid mixing, the change in turbidity was measured every 30 s for 5 min at 450 nm. The absorbance was measured continuously for 1 h at 450 nm. A unit of lysozyme activity was defined as the amount of enzyme causing a decrease in absorbance of 0.001 per min and expressed as U/mg unit.

### 2.8. Gut Histopathology

The posterior guts were excised from the collected gut samples and were fixed in 10% neutral buffered formalin for at least 48 h. The hindguts were selected and were subjected to routine tissue processing using paraffin embedded technique. Sectioning was done at 4 µm, and the tissues were stained with Hematoxylin and Eosin. These sections were examined and scored based on a previously described method [22]. Briefly, eight random fields were examined at magnifications 40× to 400×. Lesions were described and types of lesions were noted. Subsequently, these lesions were scored under the magnification of 200×. For each type of lesion, they were semi-quantitatively scored; score 0 for normal tissue, score 1 for tissue with ≤15% lesion, score 2 for tissue with 15–30% lesion, score 3 for tissue with 30–50% lesion, and score 4 for tissue with >50% lesion.

### 2.9. GALT Histomorphometric Analysis

Similar tissue sections were used for histomorphometric analysis of GALTs [23,24], which included determining GALT regions, its area size and density, in addition to the number of lymphocytes. The sections were observed using Microscope Nikon Trinocular Clinical 50 i Eclipse (Nikon, Japan) at 200× and 400× magnifications [13]. For each sample, a total of 10 microscopic fields [24], were observed and analysed. The area of GALT was measured using ImageJ [25]. Number of lymphocytes in GALTs were manually counted. Based on the number of lymphocytes and the measured area, the density of the GALT was calculated by dividing the number of lymphocytes by the area of GALT, and was expressed in cell unit µm^−2^ (Cu µm^−2^).

### 2.10. Statistical Analysis

Data of the incidence rate, gut IgM level, lysozyme activity, GALT area, lymphocyte population and GALT histomorphometric were analysed using T-test to compare between the two groups. Results were deemed significant at *p* < 0.05. All statistical analyses were performed using IBM SPSS Statistics^®^ version 25.0. 

## 3. Results

### 3.1. Clinical Findings and Gross Lesions

**Figure 2:**
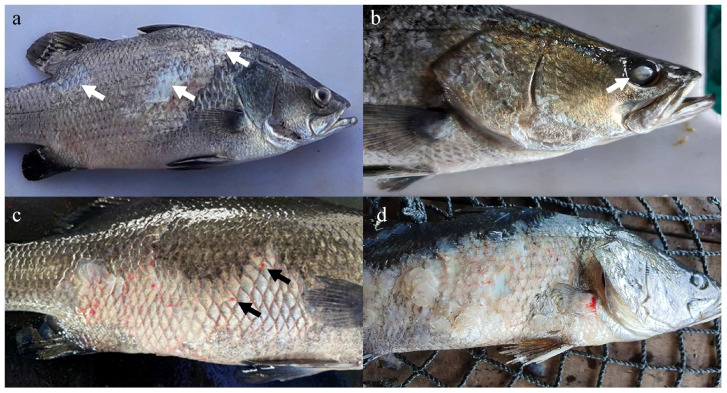
Gross lesions in Asian seabass observed throughout the 16-week vaccination trial. (**a**) Areas of scale loss (arrows), which was observed on week 2. (**b**) Ocular opacity (arrow) was observed on week 6. (**c**) Extensive loss of scale was observed on week 8. Note the reddish discoloration (arrows) due to ulceration. (**d**) Extensive loss of scale, ulceration, and fin erosion, accompanied by severe ocular opacity in week 10.

Clinical signs and gross lesions were observed in both groups. The clinical signs included abnormal swimming such as consistent swimming at the water surface, erratic swimming, and death. All clinical signs were observed as early as week 2 and continued until the end of the study. At necropsy, various gross lesions typical of vibriosis were observed in both groups between week 2 and week 16. Loss of scales was the earliest gross lesion, starting from week 2. At week 6, some fish from both groups were observed with ocular opacity, accompanied by loss of scales. In the control Group 2, these lesions were usually accompanied by skin ulceration, which was absent in the vaccinated fish of Group 1. From week 10, some fish developed fin erosion with severe ocular opacity and extensive scale loss and skin ulceration (Figure 2). These lesions persisted until the end of the experimental period. All dead fish developed at least one of these lesions.

### 3.2. Incidence Rates of Vibriosis

**Figure 3:**
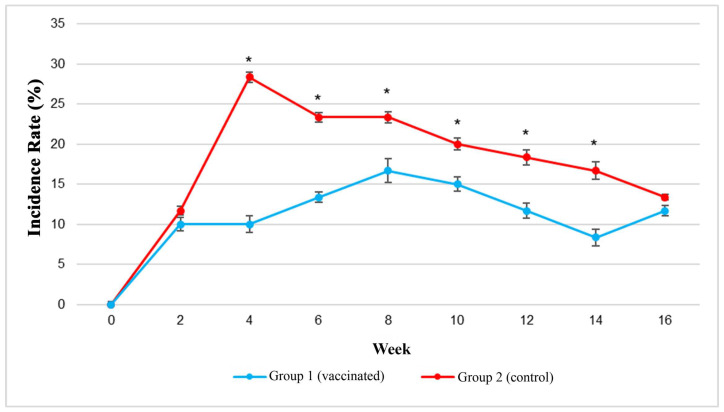
The incidence rates of vibriosis following vaccination with a feed-based vaccine. Asterisks (*) indicate significant (*p* < 0.05) difference between the two groups at various sampling points.

In week 0 both vaccinated and control groups had no incidence rate of vibriosis, while in week 2, the incidence rates of vibriosis in both groups were about 10%, showing no significant (*p* > 0.05) difference between the two groups. Thereafter, the incidence rates of vibriosis in the control group spiked and reached its highest at (28.3 ± 2.7), and remained significantly (*p* < 0.05) higher compared to the vaccinated group until week 14. On the contrary, the incidence rates of vibriosis in the vaccinated group remained lower than those of the control group from week 4 until week 14, ranging between (10–17%). In week 16, the incidence rates of vibriosis in the two groups were not significantly (*p* > 0.05) different (Figure 3). 

### 3.3. Gut Specific IgM against V. harveyi

**Figure 4:**
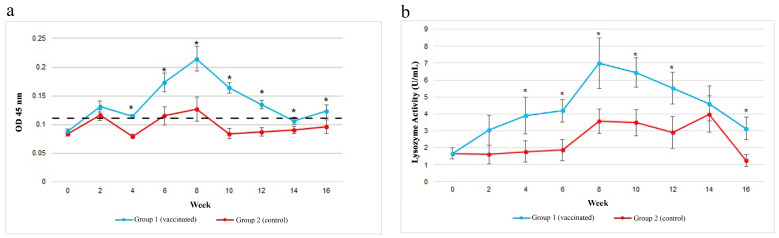
(**a**) The gut IgM response following vaccination with a feed-based vaccine. Dashed line indicates the cut-off value. (**b**) The lysozyme activity of the gut following vaccination with a feed-based vaccine. The control fish were fed commercial pellet formulated with PBS. Asterisks (*) indicate significant (*p* < 0.05) difference between the two groups at various sampling points.

In general, the gut specific IgM against *V. harveyi* of both groups showed similar patterns throughout the study. The gut IgM levels were consistently higher in the vaccinated Group 1 compared to the control Group 2 from week 2 until the end of the vaccination trial. However, significant (*p* < 0.05) higher levels were observed in the vaccinated Group 2 compared to the control Group 2 following boosters on weeks 2 and 6. In the vaccinated Group 1, Between weeks 4 and 8, the gut specific IgM against *V. harveyi* levels increased and peaked on week 8, following the second booster. Thereafter, the levels of gut specific IgM against *V. harveyi* of both groups showed a downward trend, but with those of the vaccinated Group 1 consistently showing significantly (*p* < 0.05) higher levels compared to the non-vaccinated Group 2 (Figure 4a).

### 3.4. Gut Lysozyme Activities

The lysozyme activity in the gut lavage at week 0 for both groups was similar. Then, the vaccinated group showed higher lysozyme activity in week 2. Between weeks 4 and 6, the vaccinated Group 1 showed significantly (*p* < 0.05) higher lysozyme activity than the control Group 2 and continued to increase significant (*p* < 0.05) and rapidly in week 8 following the second booster. From week 8 onwards, the lysozyme activity of the vaccinated Group 1 started to decline but remained significantly (*p* < 0.05) higher in weeks 10 and 16 than the control Group 2. The highest lysozyme activity was in week 8 and lowest was in week 0. The control group showed similar trend with slow increased between weeks 2 and 6. This was followed by a rapid increase in week 8, a slight decreased in week 10, increased back in week 14 and continued to decline in week 16. The highest lysozyme activity of the control group was in week 8 and lowest in week 0 (Figure 4b). 

### 3.5. Gut Histopathology

**Figure 5:**
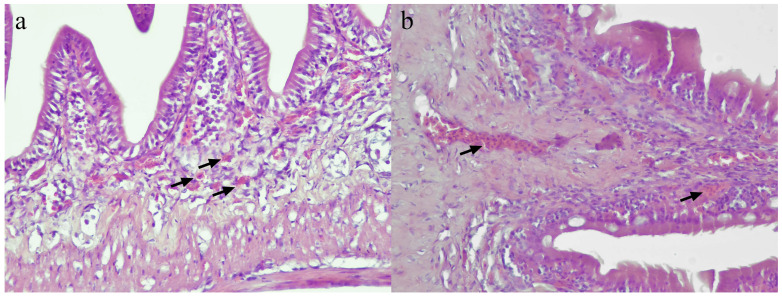
Histopathology of the gut of vaccinated and control Asian seabass. (**a**) Mild inflammation involving eosinophilic granular cell (arrows) infiltrates in the gut mucosa. (**b**) Mild congestion of blood vessels (arrows) in the lamina propria and muscularis layer.

Both groups showed similar histopathological changes in the intestine. The guts were either normal or with mild inflammation, mild congestion or minimal haemorrhage. The inflammation was characterized by mild infiltration by eosinophilic granular cells in the lamina propria, while congestion was mild, involving few blood vessels. Similarly, haemorrhage was minimal and limited to the mucosal layer (Figure 5). Histopathological scoring of the gut revealed generally low scores for all types of lesions (inflammation, congestion, and haemorrhage), and no significant (*p* > 0.05) difference was observed between the vaccinated Group 1 and the control Group 2 for all types of lesion (Table 1).

### 3.6. Assessment of GALT Regions

**Figure 6:**
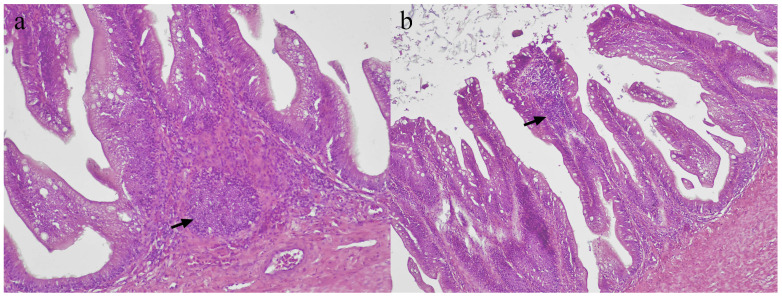
Microscopic features of GALT of Asian seabass. (**a**) GALT in the form of lymphoid aggregates (arrows) in the lamina propria at the base of intestinal villi. (**b**) GALT in the form of lymphocyte-filled villi (arrow).

GALTs were observed in the guts of both groups. They were observed either at the base of villi in the lamina propria, or within the villi, also known as lymphocyte-filled villi (Figure 6a,b). Majority of the GALT regions showed scattered pattern instead of a clear aggregation. The mean number of GALT regions of both groups was similar throughout the study period, which was generally low, ranging between 1 and 4. The number of GALT regions of both groups were constant in the first two weeks of the study. At week 4, a slight increase was observed in the control group, while the vaccinated group showed significant (*p* < 0.05) and abrupt increase in the number of GALT regions.

**Figure 7:**
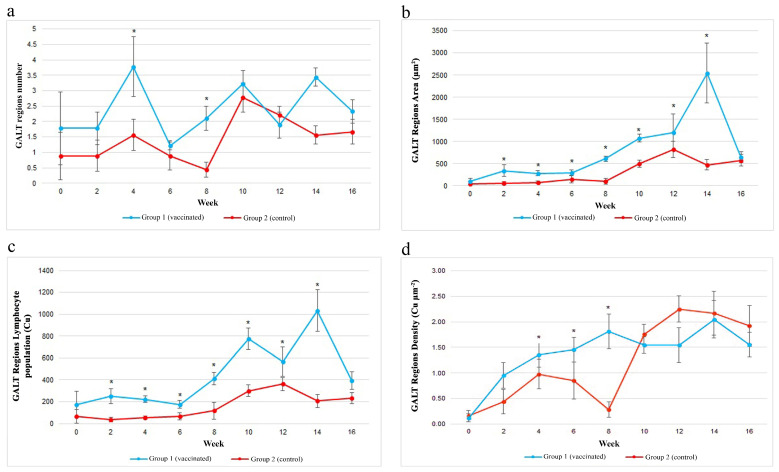
(**a**) The number of GALT regions following vaccination with feed-based vaccine. No significant (*p* > 0.05) difference was observed throughout the study. (**b**) The size of GALT regions following vaccination. (**c**) The lymphocyte population. (**d**) The density of GALT regions. The control fish were fed commercial pellet formulated with PBS. Asterisks (*) indicate a significant (*p* < 0.05) difference between the two groups at a particular sampling point.

Later, the numbers of GALT regions in both groups started to decrease before increased again in week 10. However, the number of GALT regions of the vaccinated group was significantly (*p* < 0.05) higher in week 8. Starting from week 14 onwards, the trend between the two groups were not similar (Figure 7a). It was observed that the mean number of GALT regions in the vaccinated group increased following administration of boosters at weeks 2 and 6.

Measurement and analysis of the size of GALT regions revealed that both groups had similar size of GALT regions at the beginning of the study. However, both groups showed a slow increasing trend between weeks 0 and 6. Despite the similar trend during this period, the size of GALT regions in the vaccinated group was significantly (*p* < 0.05) bigger than the control group. Between weeks 8 and 12, both groups showed marked increased in size of GALT regions. Similar to the previous weeks, both groups showed an increasing size, but the size of GALT regions in the vaccinated group was significantly (*p* < 0.05) bigger than the control group. The size of GALT regions of the vaccinated group reached peak on week 14. On the contrary, the size of GALT regions of the control Group 2 showed a slight reduction but both groups had similar size of GALT regions at the end of the study. There were significant (*p* < 0.05) differences in the size of GALT regions between both groups at all sampling points except for weeks 0 and 16 (Figure 7b).

The number of lymphocyte in the vaccinated group increased between weeks 0 and 2 that was significantly (*p* < 0.05) more than the control group. The number decreased in weeks 4 and 6 but remained significantly (*p* < 0.05) more than the control group. Following the second booster in week 6, the number of lymphocyte in the vaccinated group rapidly increased and was significantly (*p* < 0.05) more than the control group. There was a slight decrease in week 12, but recovered back in week 14 before decreased again in week 16. In weeks 12 and 14, the numbers of lymphocyte were significantly (*p* < 0.05) more than the control group. The highest number of lymphocyte was in week 14 and lowest in week 0. The control group showed a decreasing number between weeks 0 and 2, but later increased between weeks 4 and 12, decreased slightly in week 14 but finally increased back in week 16. The highest lymphocyte population was in week 12 and lowest in week 2 (Figure 7c).

**Figure 8:**
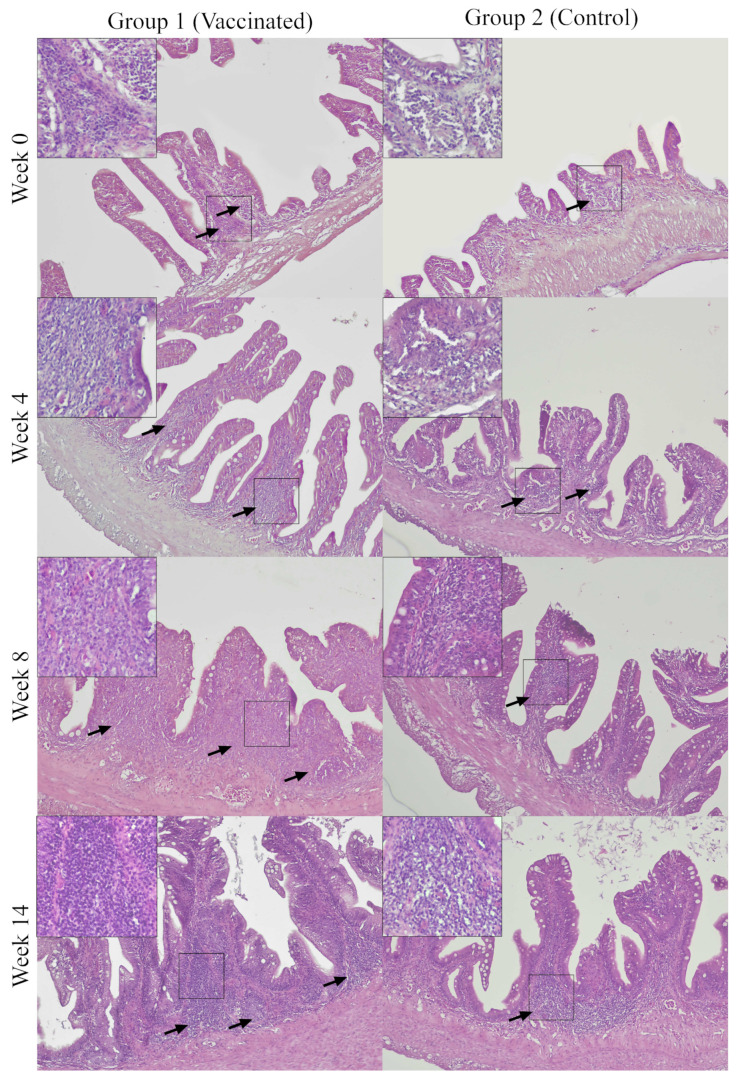
Representative micrographs of GALT regions (arrows) of the vaccinated and control Asian seabass. Insets are higher magnification for the GALT regions. GALT regions are small in both groups at week 0. GALT regions are generally larger and denser in the vaccinated Group 1 compared to the control Group 2 at week 4, 8, and 14.

The density of GALT regions in the vaccinated group showed an increasing trend between weeks 0 and 8, with rapid increase following booster vaccine at week 6. Between weeks 2 8, the vaccinated group has a significantly (*p* < 0.05) higher density of GALT regions than the control group. Weeks 10 and 12 showed a decreasing trend before increased back in week 14, and later decreased again in week 16. The highest density of GALT regions was in week 14 and lowest in week 0. The control group showed an increasing trend between weeks 0 and 4, and decreased between weeks 6 and 8, slightly increased in weeks 10 and 12, and then decreased in weeks 14 and 16. The highest lymphocyte population was in week 12 and lowest was in week 8 (Figure 7d). Micrographs of GALT regions of the vaccinated and control Asian seabass throughout the study are presented in Figure 8. 

Analysis of data throughout the 16-week study revealed a significant (*p* < 0.05) positive correlation (r = 0.88) between the size of GALT regions and number of lymphocytes in the GALT regions. Similarly, the size of GALT regions, lymphocyte population and GALT regions density showed significant (*p* < 0.05) differences between the groups but not the number of GALT regions. The GALT regions number for the vaccinated group was 2.4 ± 1.90, while that of the control group was 1.43 ± 1.49, but the mean size of GALT regions of the vaccinated group was 788.43 ± 1065.62 µm^2^ compared to 311.15 ± 389.46 µm^2^ of the control group. The lymphocyte population of the vaccinated group was 444.16 ± 407.72 Cu, which was more than the control group (160.68 ± 186.05 Cu). The GALT regions density of the vaccinated group was 1.37 ± 0.92 Cu µm^−2^, while that of the control group was 1.20 ± 1.14 Cu µm^−2^. 

## 4. Discussion

The vaccinated and non-vaccinated fish produced similar clinical signs and external lesions, which remained even after the booster vaccinations. However, the control group was generally showing a more severe skin lesion compared to the vaccinated group whereby ulceration was seen only in the control group. This observation suggests some degree of protection that hinder further development of external lesions. This is important as the presence of external lesions can reduce the marketability of fish thus, affecting the income of the farmers [2]. Vaccination enhances the resistance of fish against pathogens with the assistance of antibodies or interfering with the receptors used by the bacteria to infect cells [13]. This field study showed a significant difference on the incidence rate between the vaccinated and control groups, which reflects the ability of the vaccine to protect the vaccinated group.

Similar to a previous study on a feed-based vaccine of *Streptococcus iniae*, enhancement in the specific IgM and lysozyme activity reflects the ability of the vaccine to provide both innate and humoral responses [24]. Lysozyme activity is one of the important parameters in innate immune response, involving a mucolytic enzyme that is antibacterial, and could stimulate phagocytosis. The enzyme is known as an indicator of a non-specific immune system that can kill both Gram-negative and Gram-positive bacteria [26]. The lysozyme levels can be affected by factors like stress, infection, season, sex, sexual maturity, salinity, water temperature, pH, sedimentation, nutrition, toxicants, probiotics and immunostimulants [27]. An increase in lysozyme activity in both the control and vaccinated groups 2 weeks after the second booster reflects the innate immune response against vibriosis. There was absence of outbreak in the farm during the 16-week study but the similar increasing trends and peak between the two groups in week 8 suggests environmental changes such as an increase in water temperature that may have cause the peak which was similarly seen in a previous study done [27]. Even though, both groups showed an increasing trend, the lysozyme activity in the vaccinated group was persistently higher than the control group. Previously, a study stated that the levels of lysozyme activity will start decreasing over time because the adaptive immune response has taken over which was similarly seen in this study [27]. 

IgM in the main Ig in teleost and plays a main role in systemic immune response [3]. It is secreted by the gut epithelium [15]. The current study showed that specific IgM levels against *V. harveyi* of the gut increases after vaccination. IgM is known to be present in the gut mucus following vaccination [15]. In this study, the levels of the specific IgM against *V. harveyi* in the posterior gut remained high following the second booster till the end of the 16-week study period. This observation is generally similar to a previous study that employed a formalin-killed feed-based vaccine against *S. iniae* [24]. Following vaccination, the intraepithelial macrophages and lymphoid tissues in the gut take up the antigen. Subsequently, the antigens are phagocytosed by the macrophages before these cells migrate to other lymphoid organs, eliciting a systemic immune response [13]. In this study, the specific IgM against *V. harveyi* levels appear to be in agreement with histomorphometric of the GALT regions wherein the vaccinated fish showed stimulation of the GALT regions size, lymphocyte population, and density, but not the GALT regions number. Many previous laboratory studies have shown significant increment in the number and size of GALT regions among vaccinated fish [13,23,24]. In this field study, it is possible that the insignificant number of GALT regions between the two groups could be due to natural environmental factors. In field situations, fish are in constant contact with pathogens, where immune responses including GALT regions along the gastrointestinal tract are stimulated [28].

Significance in the numbers of GALT regions between the two groups was recorded only in weeks 4 and 8, each corresponding to the booster dose on week 2. This suggests that booster vaccinations stimulate further development of GALT [24]. In this study, various sizes and forms of GALT regions were observed; small, large, diffused, or in the form of lymphocyte-filled villi. The diffused form of GALT regions were mostly seen from week 10 onwards. It is possible that as more GALT regions were formed, they fused together, resulting in larger GALT regions but less number of GALT. This phenomenon, however, was not previously reported in fish. 

The lymphocyte population, and the size and density of GALT regions of the vaccinated fish were higher than the control fish. This reflects that vaccination recruited more lymphocytes into the lamina propria of fish, causing the GALT regions density to be higher [13]. Upon intake of vaccinated feed, the antigens translocate into the lamina propria [29], which incites of lymphocytes or other immune cells such as macrophages, plasma cells, and lymphocytes that eventually leads to formation of GALT regions [13,30,31]. In general for the teleost MALT, both B and T lymphocytes responses after an infection or vaccination, which gives rise to mucosal specific adaptive immunity in fish [32]. This is observed in this study as the vaccinated group showed an increasing trend of the GALT regions development across the 16 weeks especially for the GALT regions size and lymphocyte population following the second booster, as well as the specific IgM levels against *V. harveyi*.

Even though the parameters of the vaccinated group showed to be higher than the control, similar trends were seen in the control group for many of the measured parameters. Due to the field setting of this study, persistent contact with pathogens in the environment, and possible leaching of antigen from the vaccine due to water current are possible. Furthermore, development of GALT regions is a physiological phenomenon even in non-vaccinated fish [33]. 

## 5. Conclusions

Oral vaccination using a feed-based *Vibrio harveyi* vaccine is beneficial to caged Asian seabass where it reduces the incidence of vibriosis and the severity of external lesions. The mechanisms of this vaccine involves stimulation of the gut immunity of the fish through the production of specific IgM against *V. harveyi*, increased lysozyme activities, and stimulation of GALT regions size and density, and their lymphocyte population.

## 6. Patents

The method and composition of the oral vaccine have been filed for a patent (Patent number: PI2021000105).

## Figures and Tables

**Table 1:** Comparison of the histopathological lesion severity scores of the gut of the vaccinated and non-vaccinated control Asian seabass.

Lesions	Group 1 (Vaccinated)	Group 2 (Control)
Inflammation	0.46 ± 0.08	0.56 ± 0.10
Congestion	0.36 ± 0.07	0.30 ± 0.08
Haemorrhage	0.18 ± 0.05	0.28 ± 0.08
Overall	0.33 ± 0.04	0.38 ± 0.05

## Data Availability

The data that support the findings of the study are available on reasonable request from the corresponding author.

## References

[1] Zhang X.H., He X., Austin B. (2020). *Vibrio harveyi*: A serious pathogen of fish and invertebrates in mariculture. Mar. Life Sci. Technol..

[2] Mohamad N., Amal M.N.A., Yasin I.S.M., Zamri Saad M., Nasruddin N.S., Al-saari N., Mino S., Sawabe T. (2019). Vibriosis in cultured marine fishes: A review. Aquaculture.

[3] Ina-Salwany M.Y., Al-saari N., Mohamad A., Mursidi F.-A., Mohd-Aris A., Amal M.N.A., Kasai H., Mino S., Sawabe T., Zamri-Saad M. (2018). Vibriosis in Fish: A Review on Disease Development and Prevention. J. Aquat. Anim. Health.

[4] Deng Y., Xu L., Chen H., Liu S., Guo Z., Cheng C., Ma H., Feng J. (2020). Prevalence, virulence genes, and antimicrobial resistance of *Vibrio* species isolated from diseased marine fish in South China. Sci. Rep..

[5] Manchanayake T., Salleh A., Amal M.N.A., Yasin I.S.M., Zamri-Saad M. (2023). Pathology and pathogenesis of Vibrio infection in fish: A review. Aquac. Rep..

[6] Gomez D., Sunyer J.O., Salinas I. (2013). The mucosal immune system of fish: The evolution of tolerating commensals while fighting pathogens. Fish Shellfish Immunol..

[7] Krupesha Sharma S.R., Rathore G., Verma D.K., Sadhu N., Philipose K.K. (2011). *Vibrio alginolyticus* infection in Asian seabass (*Lates calcarifer*, Bloch) reared in open sea floating cages in India. Aquac. Res..

[8] Mohd Yazid S.H., Mohd Daud H., Azmai M.N.A., Mohamad N., Mohd Nor N. (2021). Estimating the Economic Loss Due to Vibriosis in Net-Cage Cultured Asian Seabass (*Lates calcarifer*): Evidence From the East Coast of Peninsular Malaysia. Front. Vet. Sci..

[9] Mohamad A., Zamri-Saad M., Amal M.N.A., Al-saari N., Monir M.S., Chin Y.K., Md Yasin I.S. (2021). Vaccine Efficacy of a Newly Developed Feed-Based Whole-Cell Polyvalent Vaccine against Vibriosis, Streptococcosis and Motile Aeromonad Septicemia in Asian Seabass, *Lates calcarifer*. Vaccines.

[10] Adamek M., Matras M., Rebl A., Stachnik M., Falco A., Bauer J., Miebach A.-C., Teitge F., Jung-Schroers V., Abdullah M. (2022). Don’t Let It Get Under Your Skin!—Vaccination Protects the Skin Barrier of Common Carp From Disruption Caused by Cyprinid Herpesvirus 3. Front. Immunol..

[11] Annas S., Zamri-Saad M. (2021). Intranasal Vaccination Strategy to Control the COVID-19 Pandemic from a Veterinary Medicine Perspective. Animals.

[12] Kong W.G., Qin D.C., Mu Q.J., Dong Z.R., Luo Y.Z., Ai T.S., Xu Z. (2022). Mucosal immune responses and protective efficacy in yellow catfish after immersion vaccination with bivalent inactivated *Aeromonas veronii* and *Edwardsiella ictaluri* vaccine. Water Biol. Secur..

[13] Abu Nor N., Zamri-Saad M., Md Yasin I.-S., Salleh A., Mustaffa-Kamal F., Matori M.F., Azmai M.N. (2020). Efficacy of whole cell inactivated vibrio harveyi vaccine against vibriosis in a marine red hybrid tilapia (*Oreochromis niloticus* × *O. mossambicus*) model. Vaccines.

[14] Wali A., Balkhi H. (2016). Fish vaccination and therapeutics. Int. J. Multidiscip. Res. Dev..

[15] Salinas I., Parra D., Beck B.H., Peatman E. (2015). Fish mucosal immunity: Intestine. Mucosal Health in Aquaculture.

[16] Amir-Danial Z., Zamri-Saad M., Amal M.N.A., Annas S., Mohamad A., Jumria S., Manchanayake T., Arbania A., Ina-Salwany M.Y. (2023). Field Efficacy of a Feed-Based Inactivated Vaccine against Vibriosis in Cage-Cultured Asian Seabass, *Lates calcarifer*, in Malaysia. Vaccines.

[17] Mohamad A., Mursidi F.-A., Zamri-Saad M., Amal M.N.A., Annas S., Monir M.S., Loqman M., Hairudin F., Al-saari N., Ina-Salwany M.Y. (2022). Laboratory and Field Assessments of Oral *Vibrio* Vaccine Indicate the Potential for Protection against Vibriosis in Cultured Marine Fishes. Animals.

[18] Firdaus-Nawi M., Yusoff S.M., Yusof H., Abdullah S.Z., Zamri-Saad M. (2013). Efficacy of feed-based adjuvant vaccine against *Streptococcus agalactiae* in *Oreochromis* spp. in Malaysia. Aquac. Res..

[19] Amalina N.Z., Dzarifah Z., Amal M.N.A., Yusof M.T., Zamri-Saad M., Al-saari N., Tanaka M., Mino S., Sawabe T., Ina-Salwany M.Y. (2019). Recent update on the prevalence of *Vibrio* species among cultured grouper in Peninsular Malaysia. Aquac. Res..

[20] Ismail M.S., Syafiq M.R., Siti-Zahrah A., Fahmi S., Shahidan H., Hanan Y., Amal M.N.A., Zamri Saad M. (2017). The effect of feed-based vaccination on tilapia farm endemic for streptococcosis. Fish Shellfish Immunol..

[21] Byadgi O., Uyen N.H.N., Chou R.L., Guo J.J., Lee Y.H., Lee J.W., Cheng T.C. (2018). Immunogenicity of inactivated formalin-killed *Photobacterium damselae* subsp. piscicida combined with Toll-like receptor 9 agonist in Cobia Rachycentron canadum. Aquaculture.

[22] Azzam-Sayuti M., Ina-Salwany M.Y., Zamri-Saad M., Annas S., Yusof M.T., Monir M.S., Mohamad A., Muhamad-Sofie M.H.N., Lee J.Y., Chin Y.K. (2021). Comparative Pathogenicity of *Aeromonas* spp. in cultured red hybrid tilapia (*Oreochromis niloticus* × *O. mossambicus*). Biology.

[23] Firdaus-Nawi M., Omar N., Sabri M.Y., Siti-Zahrah A., Saad M., Latifah H. (2011). The effects of oral vaccination of Streptococcus agalactiae on stimulating gut-associated lymphoid tissues (GALTs) in Tilapia (Oreochromis spp.). Pertanika J. Trop. Agric. Sci..

[24] Hayat M., Mohd Yusoff M.S., Samad M.J., Abdul Razak I.S., Md Yasin I.S., Thompson K.D., Hasni K. (2021). Efficacy of Feed-Based Formalin-Killed Vaccine of *Streptococcus iniae* Stimulates the Gut-Associated Lymphoid Tissues and Immune Response of Red Hybrid Tilapia. Vaccines.

[25] Kwan G.T., Finnerty S.H., Wegner N.C., Tresguerres M. (2019). Quantification of Cutaneous Ionocytes in Small Aquatic Organisms. Bio-Protocol.

[26] Biller J.D., Polycarpo G.D.V., Moromizato B.S., Sidekerskis A.P.D., Silva T.D.D., Reis I.C.D., Fierro-Castro C. (2021). Lysozyme activity as an indicator of innate immunity of tilapia (*Oreochromis niloticus*) when challenged with LPS and *Streptococcus agalactiae*. Rev. Bras. Zootec..

[27] Saurabh S., Sahoo P.K. (2008). Lysozyme: An important defence molecule of fish innate immune system. Aquac. Res..

[28] Yu Y.Y., Ding L.G., Huang Z.Y., Xu H.Y., Xu Z. (2021). Commensal bacteria-immunity crosstalk shapes mucosal homeostasis in teleost fish. Rev. Aquac..

[29] Hayat M., Sabri M.Y., Intan-Shameha A.R., Ina-Salwany M.Y., Thompson K.D. (2020). Localisation of antigens in the gut post-challenge with *Streptococcus iniae* in vaccinated and non-vaccinated red hybrid tilapia (*Oreochromis* sp.). Aquac. Int..

[30] Attaya A., Secombes C.J., Wang T. (2020). Effective isolation of GALT cells: Insights into the intestine immune response of rainbow trout (*Oncorhynchus mykiss*) to different bacterin vaccine preparations. Fish Shellfish Immunol..

[31] Parra D., Korytář T., Takizawa F., Sunyer J.O. (2016). B cells and their role in the teleost gut. Dev. Comp. Immunol..

[32] Salinas I. (2015). The Mucosal Immune System of Teleost Fish. Biology.

[33] Firdaus-Nawi M., Zamri-Saad M., Nik-Haiha N.Y., Zuki M.A.B., Effendy A.W.M. (2013). Histological assessments of intestinal immuno-morphology of tiger grouper juvenile, *Epinephelus fuscoguttatus*. SpringerPlus.

